# Clinical pharmacist prescriber in primary care in Slovenia: prospective non-randomised interventional study focused on clinical outcomes and quality of life

**DOI:** 10.3389/fphar.2025.1690480

**Published:** 2025-09-15

**Authors:** Matej Stuhec, Alenka Kovacic, Marjetka Korpar, Ana Banovic Koscak, Barbara Koder, Dunja Mahoric, Spela Bernik Golubic, Eva Gorup Cedilnik, Vesna Homar, Aleksandar Stepanovic, Danica Rotar Pavlic

**Affiliations:** ^1^ Department of Pharmacology and Department of Clinical Pharmacy, Medical Faculty Maribor, University of Maribor, Maribor, Slovenia; ^2^ Department of Clinical Pharmacy, Ormoz’s Psychiatric Hospital, Ormoz, Slovenia; ^3^ Murska Sobota General Hospital, Murska Sobota, Slovenia; ^4^ Lekarne Ptuj, Ptuj, Slovenia; ^5^ Goriška lekarna Nova Gorica, Nova Gorica, Slovenia; ^6^ Gorenjske lekarne, Kranj, Slovenia; ^7^ Lekarna Toplek, Ptuj, Slovenia; ^8^ Slovene Chamber of Pharmacy, Ljubljana, Slovenia; ^9^ University of Ljubljana, Faculty of Medicine, Department of Family Medicine, Ljubljana, Slovenia

**Keywords:** pharmacist prescriber, clinical pharmacy, family medicine, medicationreview, primary care

## Abstract

**Introduction:**

Clinical pharmacist prescribers in primary care settings and their impact on patient-reported outcomes (PROs) and clinical outcomes have not been described outside English-speaking countries.

**Aim:**

The aim of this prospective interventional pilot study was to assess the impact of pharmacist prescribers on clinical results and patient-reported outcomes (PROs), while describing their development, evaluation, and implementation in Slovenia.

**Methods:**

This prospective, 6-month, interventional, non-randomised study started in November 2024 and concluded in June 2025 in four primary care settings in Slovenia. Clinical pharmacists reviewed medications of patients and additionally prescribed medications based on the Collaborative Practice Agreement (CPA). In this process, they cooperated with patients and general practitioners (GPs). Only patients with an established diagnosis for selected non-communicable chronic conditions were included. The primary outcomes were changes in PROs, including quality of life (assessed via EQ-5D-VAS), and the Medication Appropriateness Index (MAI). Secondary outcomes included the prescription acceptance rate by GPs (percentage) and adherence to treatment guidelines. Tertiary outcomes involved the number of prescriptions that met the predefined clinical outcomes.

**Results:**

The study included 119 patients, with a mean age of 72.3 years (SD = 10.0). Quality of life improved from 63.6/100 (SD = 18.7) at baseline to 71.4/100 (SD = 15.9) at the end of the study (p = 0.000), with a corresponding QALY difference of 0.0252. The effect size (Cohen’s d) was 0.448 (95% CI: 0.084 to 0.812. The number needed to treat (NNT) was 4.0. During the study, clinical pharmacists prescribed 264 prescriptions to 119 patients, resulting in an acceptance rate of 91.3%. Adherence to treatment guidelines improved significantly (29.8% vs. 90.9%; p = 0.000). The effect size, expressed as an odds ratio (OR), was 25.7 (95% CI: 15.6–42.4). The number of prescriptions achieving the predefined clinical outcomes was significantly higher at the end of the study (70.8% vs. 6.4%; p = 0.000), with an OR of 33.9 (95% CI: 19.1–60.4). Deprescribing accounted for 25.3% of all protocols.

**Conclusion:**

This study demonstrates that prescriptions made by clinical pharmacists in collaboration with GPs, as specified in the CPA, improved PROs and clinical outcomes for predefined conditions.

## 1 Introduction

Polypharmacy represents a significant burden in Europe and is highly prevalent, particularly among elderly patients in primary care. It often leads to poorer clinical outcomes and increased healthcare costs (Bennie et al., 2024; Maher et al., 2014; Midão et al., 2018). Two systematic reviews found a high prevalence of polypharmacy in primary care across Europe, raising concerns about psychotropic polypharmacy, particularly the notably high use of benzodiazepines in this population (Bennie et al., 2024; Midão et al., 2018).

A study involving 503 patients in general practice in Slovenia also demonstrated high polypharmacy use among the elderly (Gorup and Šter, 2017). Additionally, Maher et al. reported that approximately 50% of older adults (≥65 years) were prescribed at least one unnecessary medication. They identified a strong relationship between polypharmacy and adverse clinical outcomes and recommended increased collaboration with clinical pharmacists to promote rational medication use in this population (Maher et al., 2014).

In addition to medication-related problems, chronic conditions are a critical factor in optimizing medication management in primary care (Kessler et al., 2003; Lech et al., 2022). Despite significant efforts by general practitioners (GPs) and other healthcare professionals, there remains considerable room for improvement (Kessler et al., 2003; Lech et al., 2022; Smolders et al., 2009; Redon et al., 2016). Kessler and colleagues found that fewer than 50% of patients with depression in primary care receive adequate treatment (Kessler et al., 2003). Similar findings were reported in the Netherlands, where adherence to treatment guidelines for depression was only 42%, and for hypertension, only 40% of patients achieved adequate blood pressure control (Smolders et al., 2009; Redon et al., 2016).

Research on adherence to treatment guidelines for diabetes in primary care showed that only 56% of patients follow recommended management protocols (Alliabi et al., 2022). GPs often report poor communication with other specialists when managing chronic conditions. In Germany, most GPs indicated inadequate communication with psychiatrists, despite GPs being responsible for diagnosing and managing the majority of depression cases in primary care (Lech et al., 2022). In many countries, including Slovenia, the limited number of GPs may contribute to the suboptimal management of chronic diseases (European Health Information Gateway).

Collaboration between GPs and clinical pharmacists is an important strategy for reducing medication-related problems and optimizing treatment (Stuhec, 2021; Urbańczyk et al., 2023). This collaboration can involve both pharmacist prescribers and non-prescribers, with clinical pharmacists providing services such as medication reviews and medication reconciliation, which are nationally accepted and reimbursed in some countries (Stuhec, 2021; Urbańczyk et al., 2023). Medication reviews in general practice have been extensively studied in countries where they are already reimbursed, including the United Kingdom, the United States, and Slovenia (Stuhec, 2021; Komwong et al., 2018; Chisholm-Burns et al., 2010).

Unfortunately, in most Central European countries, such services are not reimbursed in general practice, with the exception of Slovenia. In Slovenia, clinical pharmacists conduct medication reviews—specifically, type 3 (advanced) medication reviews as defined by the Pharmaceutical Care Network Europe (PCNE)—on behalf of GPs with a referral paper. Since 2017, these services have been reimbursed by the national insurance. Research has demonstrated a positive impact, including reductions in polypharmacy, drug-drug interactions (DDIs), potentially inappropriate medications (PIMs), improved adherence to treatment guidelines, and enhanced quality of life (Stuhec, 2021; Urbańczyk et al., 2023). In Slovenia, clinical pharmacists provide medication reviews in almost all general practices within primary care ambulatory settings, representing a powerful approach for medication management. This collaboration supports GPs in managing polypharmacy and improving patient outcomes (Stuhec, 2021; Urbańczyk et al., 2023).

A pharmacist prescriber represents an additional step beyond the previously mentioned medication review. It has been well developed and implemented in the United Kingdom, where clinical pharmacists have been prescribing independently within their competencies. In New Zealand and the United States, clinical pharmacists collaborate as dependent prescribers, with the United States requiring a collaborative practice agreement (CPA) for such collaboration (Choe et al., 2018; American Pharmacists Association (APhA); Raghunandan et al., 2021; Carter et al., 2024). In the United Kingdom, there are over 2,000 pharmacist-independent prescribers in general practice, a development that began in 2006 from the earlier supplementary prescriber model. Pharmacists require additional education and training provided by approved organisations (Tonna et al., 2007; Cope et al., 2016). The role of pharmacist prescribers has been extensively researched in the United Kingdom, particularly in prescribing and deprescribing (Tonna et al., 2007; Alharthi et al., 2023).

A meta-analysis of 46 studies (37,337 participants) compared non-medical prescribing by nurses and pharmacists with standard care (Weeks et al., 2016). There was moderate certainty of evidence for studies assessing blood pressure at 12 months (mean difference (MD) −5.31 mmHg, 95% confidence interval (CI) −6.46 to −4.16; 12 studies, 4,229 participants) and low-density lipoprotein (LDL) cholesterol (MD -0.21, 95% CI -0.29 to −0.14; 7 studies, 1,469 participants). High certainty evidence was found for glycated haemoglobin management (HbA1C) at 12 months (MD -0.62, 95% CI -0.85 to −0.38; 6 studies, 775 participants) (Weeks et al., 2016). The authors also compared prescribing by pharmacists to that by nurses (only one study), which showed substantial improvements in both groups after 6 months: 43.4% of participants in the pharmacist case manager group met both systolic blood pressure and LDL cholesterol target guidelines, compared with 30.9% in the nurse-led group (an absolute difference of 12.5%; number needed to treat = 8, p = 0.03) (McAlister et al., 2014). In a recent scoping review, encompassing 63 studies, researchers focused on non-medical prescribing, including pharmacists and nurses involved in mental health management in primary care. Both pharmacists and nurses prescribe antidepressants widely, though their practices differ. The authors concluded that more qualitative research is needed while the role is positive (Alsaeed et al., 2025).

In New Zealand, clinical pharmacists prescribe the most medications for infections and pain. The authors noted that around 50% of GPs are expected to retire within the next 10 years, creating opportunities for the development of pharmacist prescribers (Raghunandan et al., 2021). In New Zealand, clinical pharmacists can prescribe most medications as GPs do; no special CPA document is necessary, as in the US (Pharmacist Prescriber Scope of Practice). Similar developments are occurring in Canada and Australia; however, collaboration within CPA and/or community pharmacies remains limited and has not yet been implemented nationally (Nakhla et al., 2024).

The impact of clinical pharmacists in the medication review process has been extensively studied. Still, the development, implementation, and evaluation of pharmacist prescribers in general practice nationally have not been described outside the United Kingdom, United States, Canada, and New Zealand. Developing models and conducting pilot trials are essential steps for successful implementation.

In this context, we describe the development, evaluation, and implementation of pharmacist prescribers in Slovenia through a 6-month prospective interventional study. We hypothesise that this collaboration will positively affect patient-reported outcomes (PROs), adherence to treatment guidelines, and improve clinically predefined outcomes.

## 2 Materials and methods

### 2.1 Settings

This study was conducted at four primary care settings in Slovenia, located in both Slovenian cohesion regions: Ormoz, Nova Gorica, Ptuj, and Jesenice. In these settings, clinical pharmacists (also referred to as pharmacist consultants) provide medication reviews (specifically, type 3 or advanced medication reviews) on behalf of GPs, using a referral paper. (Stuhec, 2021; PCNE). Type 3 (advanced) medication reviews are based on a patient’s history, relevant patient information, and clinical data. They address all critical aspects outlined by the PCNE, including drug-drug interactions, side effects, unusual dosages, adherence issues, drug-food interactions, effectiveness concerns, over-the-counter medication problems, unindicated medications, missing indications, and dosage issues (Stuhec, 2021; PCNE; Stuhec et al., 2019a).

Clinical pharmacists work daily in Slovenia in primary care settings alongside GPs and have access to complete patient records, including lab test results. Participating clinical pharmacists have established a strong collaborative relationship with GPs in these primary care settings and those serving the surrounding region, including nursing home settings (Stuhec, 2021).

In Slovenia, clinical pharmacists do not have prescription authority; instead, they recommend GPs based on their medication reviews. GPs then review the medication recommendations and make the final decision (Stuhec, 2021). Medication reviews conducted by clinical pharmacists are also recognized as pharmaceutical services under Slovenian legislation (Slovenian Pharmacy Act 2016) (Stuhec, 2021).

### 2.2 Development

Slovenia has a relatively low number of GPs compared to Western countries, a high percentage of elderly patients, and consequently, a high prevalence of polypharmacy (Stuhec, 2021). Additionally, family medicine and clinical pharmacy are well-developed fields at the European level, creating an opportunity for pilot trial development that promotes collaboration rather than competition between the two specialties (Stuhec, 2021).

This reimbursed service, medication review in Slovenia, served as the foundation for this study. We expanded the medication review service to include pharmacist prescribers with additional monitoring. Furthermore, the Ministry of Health of the Republic of Slovenia confirmed that a pilot trial of this collaboration was necessary (October 2023). This endorsement marked a significant milestone for the initiation of the pilot. With support from the Ministry of Health, the Health Insurance Institute of Slovenia, and the Slovene Chamber of Pharmacy, the Slovenian Professional College of Family Medicine agreed to participate in the pilot trial. The Association of Patient Organisations of Slovenia also expressed support through a letter of endorsement.

The joint working group consists of representatives from the Slovene Chamber of Pharmacy—clinical pharmacist specialists with experience in pilot trials—and representatives from the Slovenian Professional College of Family Medicine—family medicine specialists with pilot trial experience. In May 2024, the Ministry of Health announced a call for a research grant titled “Examining the Benefits and Risks of Dependent Prescribing Practice in the Context of Pharmaceutical Care,” with a main funding amount of 70,000 EUR for 1 year. The project’s aim was to assess the feasibility of expanding the current collaboration to include dependent prescribing in pharmacist-led clinics (evaluating treatment outcomes for predefined disease states) and to identify the systemic and legislative changes necessary to incorporate this new pharmaceutical competence into the Slovenian healthcare system. The working group applied for the grant through their affiliations: the Medical Faculty Maribor (University of Maribor) and the Medical Faculty Ljubljana (University of Ljubljana). They were successful and received funding from October 2024 to September 2025.

The joint working group, consisting of representatives from the Slovene Chamber of Pharmacy and the Slovenian Professional College of Family Medicine, prepared all necessary protocols, including the CPA document (available in Supplementary Data sheet 3) and protocols for the included medical conditions, including predefined outcomes (Supplementary Data sheet 4). GPs could authorize clinical pharmacists to prescribe either a single medication for a specific condition or all medications permitted for prescribing in Slovenia (as specified in the CPA). The participating GPs had the discretion to select these options. Clinical pharmacists could prescribe only after GPs confirmed diagnoses, and prescriptions had to be digitally signed by the GPs before dispensing (no emergency or acute prescriptions). Patients and GPs could withdraw from the collaboration at any time during the study.

Five clinical pharmacists with primary care experience were invited to participate in the trial. Each has more than 5 years of experience conducting medication reviews in primary care settings, which was essential for the pilot’s success, given their established collaborative relationships.

The working group established primary outcomes for each condition. Each protocol includes a 6-month target value, a measurement scale, and relevant clinical guidelines. The focus was on clinical outcomes rather than solely medication-related problems and polypharmacy; therefore, specific outcomes and target values were defined in each protocol based on relevant guidelines (e.g., depression remission, target HbA1c levels). The CPA and protocols were also discussed with GPs from the practice and were further improved during the implementation process. The protocols and CPA document were finalized and presented to all clinical pharmacists and GPs before the pilot started. The CPA addresses ten main medical conditions (10 protocols), which are summarised in Table 1. All protocols had the same format, including target outcomes, guidelines and recommendations. We have attached four protocols, which were the most frequently used, in Supplementary Material-Data sheet 4 (protocols for arterial hypertension, dyslipidemia, depression, and deprescribing).

**TABLE 1:** Review of protocols, including predefined clinical outcomes and prescription authority.

Protocol number	Protocol	Predefined clinical outcomes based on	Prescription authority
1	Lipids not in target range (dyslipidemia diagnosis)	S-LDL target value	Initiation, Adjustments, Discontinuations
2	Neuropathic pain—therapy adjustment (neuropathic pain diagnosis)	Visual Analogue Scale [VAS] target score	Initiation, Adjustments, Discontinuations
3	Blood pressure not in target range (arterial hypertension diagnosis)	Blood pressure in mmHg target value	Initiation, Adjustments, Discontinuations
4	Diabetes—HbA1c not in target range (type II diabetes diagnosis)	HbA1c target value	Initiation, Adjustment, Discontinuation (only oral medications)
5	Depression remission not achieved (unipolar depression diagnosis)	Depression remission (Patient Health Questionnaire-9 [PHQ-9] target score)	Initiation, Adjustment, Discontinuation
6	Use of anti-dementia drugs (Alzheimer’s dementia diagnosis)	Mini-Mental State Examination (MMSE) target score	Initiation, Adjustment, Discontinuation
7	Gout treatment (gout diagnosis)	Uric acid level target value	Initiation, Adjustment, Discontinuation
8	Adjustments based on renal and hepatic function (renal and/or hepatic insufficiency)	Adjustments according to the Summary of the Product Characteristics	Initiation, Adjustment, Discontinuation
9	Deprescribing to optimize therapy	Priscus list criteria adherence, Medications without indication	Adjustment, Discontinuation
10	Titration of asthma medications (asthma diagnosis)	Asthma Control Test target score	Adjustment only
Out of the protocol	Only medications specified by the general practitioners (GPs) in the CPA document were allowed— (medications that GPs in Slovenia can prescribe autonomously)		

In 2024, the Slovenian National Medical Ethics Committee granted ethical approval (16 October 2024; N#0120-330/2024-2711-3), allowing the study to commence.

The Ethical Approval application, which the working group prepared and approved, included patient information, including consent forms. The consent form was approved by the Slovenian National Medical Ethics Committee, and only patients who signed the consent form were included in the study. Patients who did not sign the consent form received only a medication review without prescribing by clinical pharmacists.

The Ministry of Health of the Republic of Slovenia compensates participants, including clinical pharmacists, GPs, and researchers (grant number V3-24041).

### 2.3 Type of intervention and implementation

This prospective, interventional, non-randomized study started in November 2024 and concluded in June 2025. Clinical pharmacists provided recommendations within the medication review and additional prescribing after each patient visit. The project was introduced at the outset in four different primary care settings. Before participating, every patient provided informed consent, and only those who signed the consent form were included. Clinical pharmacists conducted medication reviews and prescribed additional medications in a CPA, confirming the second appointment. They could prescribe medications on behalf of the GP, who specified which medication groups the pharmacists were authorized to prescribe in a CPA.

Throughout the study, clinical pharmacists monitored patients from the enrollment to the end of the pilot trial. Each patient was assessed at least three times: at enrollment (baseline), the second visit (after 2 months), and the last visit (6 months after the first). Pharmacists could contact patients more frequently, if necessary, but outcomes were only recorded at these three main points. Pharmacists prescribed medications in the same way GPs prescribe in Slovenia. Prescriptions were entered into the eSystem and confirmed by GPs, enabling drug dispensation at community pharmacies. GPs were required to specify the reason for any non-acceptance within the eSystem. Medication reviews were documented in the primary care setting’s eSystem. Each patient received three medication reviews, and prescriptions were recorded within the eSystem, allowing GPs to confirm, modify, or reject the prescriptions. The patient was informed about the next visit through the e-Invitation or by the care setting’s informant. Clinical pharmacists also notified patients that their prescriptions would be ready at the pharmacy in a few days. All five clinical pharmacists included in the pilot completed medication reviews using the standardized form approved by the Slovene Chamber of Pharmacy (Stuhec, 2021). Clinical pharmacists provided recommendations within the medication review and additional prescribing after the patient’s visit.

### 2.4 Inclusion and exclusion criteria

Selection criteria were based on GP referrals. Only patients with an established diagnosis for various conditions were included. GPs primarily referred patients with some pharmacological issues, such as untreated conditions or variations in achieving target outcomes. Therefore, the study population was not focused on patients with polypharmacy, who are typically included in medication reviews in Slovenia. Instead, the working group concentrated on conditions that had not yet been treated and their clinical outcomes for established diagnoses (e.g., depression remission). The study included patients from all primary care settings involved in the pilot trial. Referrals were made solely based on the GP’s referral paper, which included the CPA document. Both the patient and the GP had to sign the CPA. The population consisted of all patients referred to the clinical pharmacist. Each patient was included in the study only once.

The inclusion criterion was that the clinical pharmacist had made at least one suggestion to modify the therapy during the medication review (at least one prescription including deprescription). Only patients for whom all three medication reviews were completed and who completed the entire study period were included in the final analysis.

### 2.5 Outcomes

The patients’ key characteristics (age, gender, number of medications, drug-drug interactions [DDIs], and potentially inappropriate medications [PIMs]) were assessed. DDIs were identified using the Lexicomp Online^®^ database and categorized as X-type (contraindicated) and D-type (major). To identify PIMs in elderly patients, we referred to the latest Priscus List 2.0 (Mann et al., 2023). We also included the number of patients’ visits to GPs’ settings in the 3 months before and after the first medication reviews. Drug-related problems (DRPs) were categorized according to the Slovenian classification of drug-related problems (DRP-SLO-V1) (Horvat and Kos, 2016).

The primary outcomes were changes in PROs. PROs evaluated included quality of life (assessed via EQ-5D-VAS), quality-adjusted life years (QALYs), and the Medication Appropriateness Index (MAI). Effect size (Cohen’s d) for differences in quality of life based on EQ-5D-VAS between study points and 95% confidence intervals (CIs) was calculated. The MAI was assessed during the study, excluding question N#10 (cost-effectiveness). Utility scores were derived from EQ-5D-VAS scores. Differences in QALYs were calculated using the trapezoidal rule. The Anticholinergic Burden score was also calculated using the Anticholinergic Burden Calculator (Hanlon et al., 1992; Anticholinergic Burden Calculator).

The secondary outcomes included the prescription acceptance rate by GPs (%), the description of prescriptions provided by clinical pharmacists, and adherence to treatment guidelines for the defined conditions. The effect size, expressed as odds ratios (OR) with 95% confidence intervals (CI), was calculated for adherence to treatment guidelines.

The tertiary outcomes were focused on predefined clinical outcomes. They included describing the protocols prescribed by clinical pharmacists, the acceptance rate (%), and the change in the number of prescriptions reaching the predefined clinical outcomes (end/baseline). The effect size as OR, with 95% CI, was calculated for adherence to the predefined clinical outcomes. These outcomes included: diabetes management (HbA1c and blood glucose), lipid levels (LDL-C), neuropathic pain (Visual Analogue Scale [VAS]), depression remission (Patient Health Questionnaire-9 [PHQ-9]), controlled blood pressure (measured in mmHg), cognitive function (Mini-Mental State Examination [MMSE]), gout (uric acid level), renal and hepatic dose adjustments (based on the Summary of Product Characteristics), asthma control (Asthma Control Test), and deprescribing (Priscus list). Based on the latest treatment guidelines, the working group confirmed target values for all outcomes included in the protocols before the study. It incorporated them into the pharmacists’ prescriber protocols.

In addition to patient-reported outcomes (PROs) and clinical outcomes, we assessed the impact of pharmaceutical interventions on reducing treatment costs by conducting a simple cost-benefit analysis (CBA). Since Slovenian data were not available, we used data on the financial values of individual pharmacist interventions from a study by Lee et al., conducted in the United States (Lee et al., 2002). They included the following interventions: discontinuation of X DDI, dose adjustments, discontinuation of duplication in therapy, initiation of medication for an untreated condition, discontinuation of medication without an approved indication, and other interventions such as drug discontinuation and initiation. These values were then adjusted to 2025 prices using the CPI Inflation Calculator. Total costs for medication reviews during the study were calculated based on data from the Health Insurance Institute of Slovenia: EUR 59 for the first medication review and EUR 41.3 for subsequent reviews; for patients on 10 or more medications, the cost was double that of EUR 59.

### 2.6 Data collection

Data collection began after the study started. The Working Group prepared a Microsoft Excel 2016 worksheet, which the researchers used to record the data. Data were collected from patients’ medical records, Slovenian central digital prescription registry, and the eSystem across primary care settings. The three main research points corresponded to three medication reviews: at enrollment (time 0), after 2 months (first review), and after 6 months (second review). To ensure anonymity, we encrypted data for patients, clinical pharmacists, and GPs. Information about prescriptions was obtained from the eSystem and patients’ charts.

Four researchers (M.K., A.B., M.S., B.K.), all experienced clinical pharmacists and researchers, collected and extracted data from November 2024 to June 2025. M.S. primarily conducted statistical analysis, and all authors approved the results.

Other researchers contributed to various aspects of the study, including reviewing, interpretation, and providing external review to help minimize bias.

### 2.7 Statistics

We used descriptive statistics to summarize the main characteristics of the study. Numerical results were expressed as sums, with standard deviations (SD), and minimum and maximum values where applicable. The Kolmogorov-Smirnov test was employed to assess normality. Based on the results, different statistical tests were selected: paired samples t-tests for normally distributed variables and the Wilcoxon signed-rank test for non-normally distributed variables.

The sample size was determined based on previous studies conducted in primary care settings, including a similar pilot trial in Ireland (Stuhec, 2021; Stuhec et al., 2019a; Cardwell et al., 2020) and a power analysis performed using G*Power^®^ software. With an alpha level of 0.05, a power of 0.90 (1–β), and an effect size of 0.3, the calculated total sample size required was 115, accounting for a 20% attrition rate. Additionally, the Bonferroni correction was applied to adjust for multiple comparisons, setting a significance level of p < 0.05, which was modified to the adjusted p-value.

Effect sizes for continuous variables were calculated using Cohen’s d, while for categorical variables, odds ratios (ORs) with 95% confidence intervals (CIs) and number needed to treat (NNT) were determined using Psychometrica^®^. Data analysis was performed using Microsoft Office Excel^®^ 2016 and IBM SPSS Statistics version 26. This study adhered to the STROBE (Strengthening the Reporting of Observational Studies in Epidemiology) guidelines (von Elm et al., 2008). The EuroQol Group approved using EQ-5D-VAS for study purposes in November 2024.

## 3 Results

### 3.1 General results

This study included 126 patients who received medication reviews from clinical pharmacists, including prescribing based on the CPA document. In this study, 23 GPs participated and referred patients to clinical pharmacists. Four of five clinical pharmacists completed the study because one of them left and did not continue the project due to severe health problems. Of these, 119 patients with a mean age of 72.3 years (SD = 10.0) were eligible for the final analysis due to complete data sets (94.5%). Men accounted for 51.3% (N = 61) of participants, women for 48.7% (N = 58). The mean age of the patients at baseline was 72.3 years (SD = 10.0), and they had an average of 7.68 diagnoses (SD = 3.9).

**FIGURE 1:**
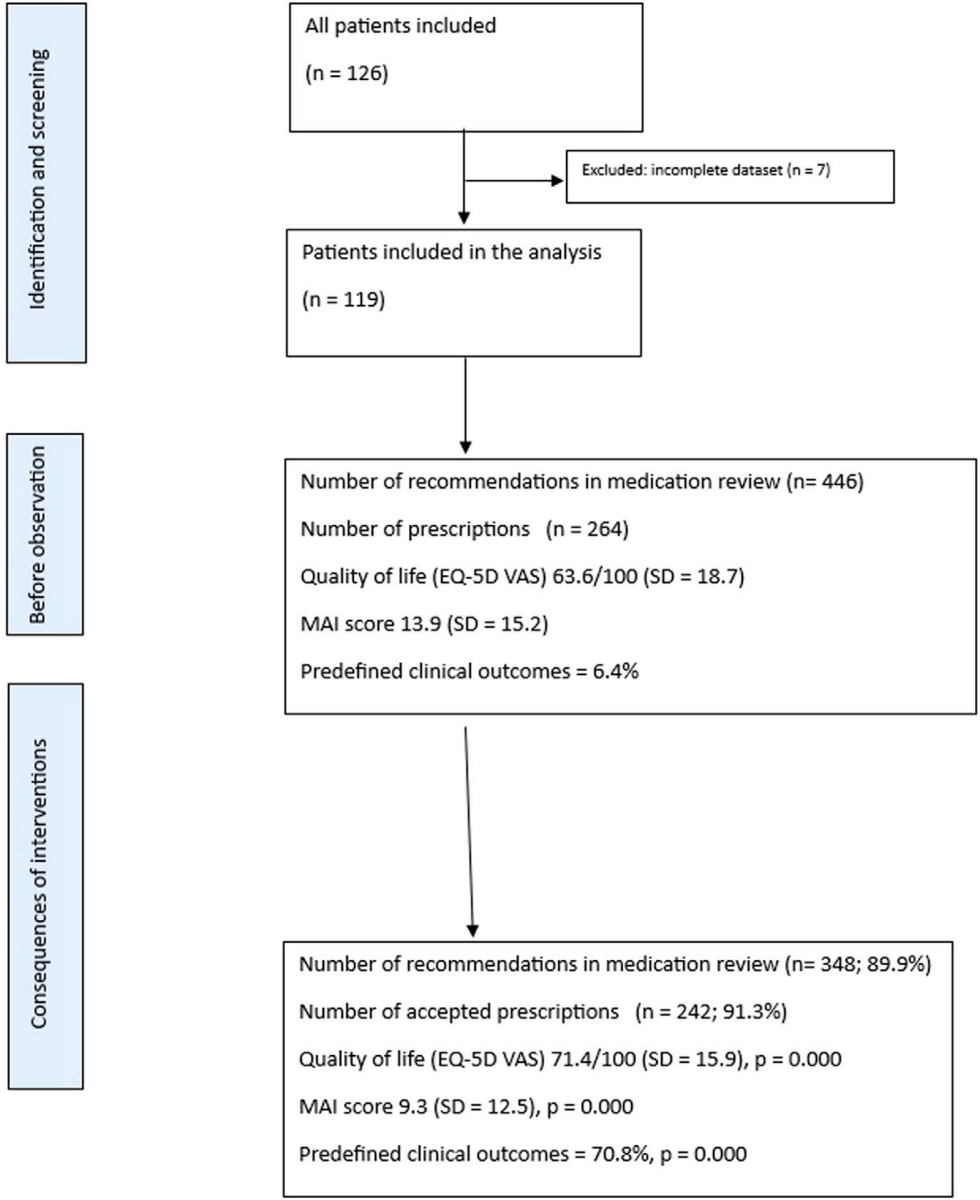
Flowchart.

On average, patients were prescribed 9.85 (SD = 4.8) medications at baseline, 10.2 (SD = 4.6) after the second visit, and 10.0 (SD = 4.5) medications at the end of the pilot trial. Clinical pharmacists provided 446 recommendations during medication reviews (mean per review: SD = 1.8; maximum 10, minimum 1) in the first medication review. GPs accepted 387 of these recommendations, resulting in an acceptance rate of 86.7%, and 348 recommendations were continued until the end of the study (89.9%). The Flowchart is presented in Figure 1.

At the baseline, patients had an average of 16 X-type DDIs (0.13 per patient, SD = 0.43) and 132 D-type DDIs (1.12 per patient, SD = 1.2). The number of X-type DDIs decreased non-significantly to 9 (0.08 per patient, SD = 0.350; p = 0.71). All X-type DDI interactions persisted until the end of the study. The number of D-type DDIs was reduced to 99 after the second visit (0.83 per patient, SD = 1.195) and increased slightly to 100 by the end of the study. The difference between the baseline and the last visit was statistically significant (p = 0.013), as was the difference between the baseline and the first visit (p = 0.009).

At baseline before medication review, patients had an average of 180 PIMs listed in Priscus (mean 1.86, SD = 1.6). This number decreased significantly following medication review, 138 PIMs (mean 1.42, SD = 1.40; p = 0.000) and further reduced to 128 (mean 1.32, SD = 1.09; p = 0.000) by the end of the study.

### 3.2 Primary outcomes

**FIGURE 2:**
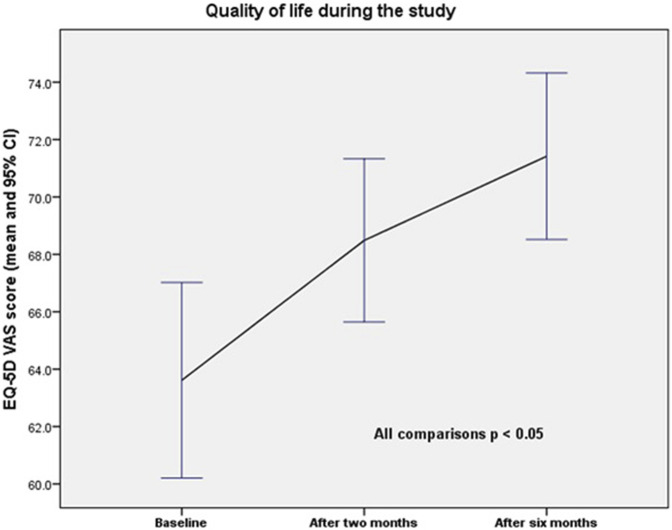
Quality of life during the study using EQ-5D VAS score.

One hundred and nineteen patients were included in the quality of life study, as well as the MAI and anticholinergic Burden scores. Quality of life, according to the EQ-5D VAS, increased from 63.6/100 (SD = 18.7) at baseline to 68.5/100 (SD = 15.7) after the second visit, and to 71.4/100 (SD = 15.9) after the end of the study. The differences were statistically significant (baseline vs. 2 months; p = 0.001; baseline vs. 6 months; p = 0.000; and 2 months vs. 6 months; p = 0.002). The changes in quality of life over the study period are presented in Figure 2.

The calculated difference in QALYs was 0.0252 between baseline and the end of the study, 0.00408 between baseline and 2 months, and 0.02117 between 2 months and the end of the study. The effect size (Cohen’s d) was 0.448 between baseline and the end of the study, with a 95% confidence interval (CI) of 0.084–0.812. The number needed to treat (NNT) was 4.0.

**FIGURE 3:**
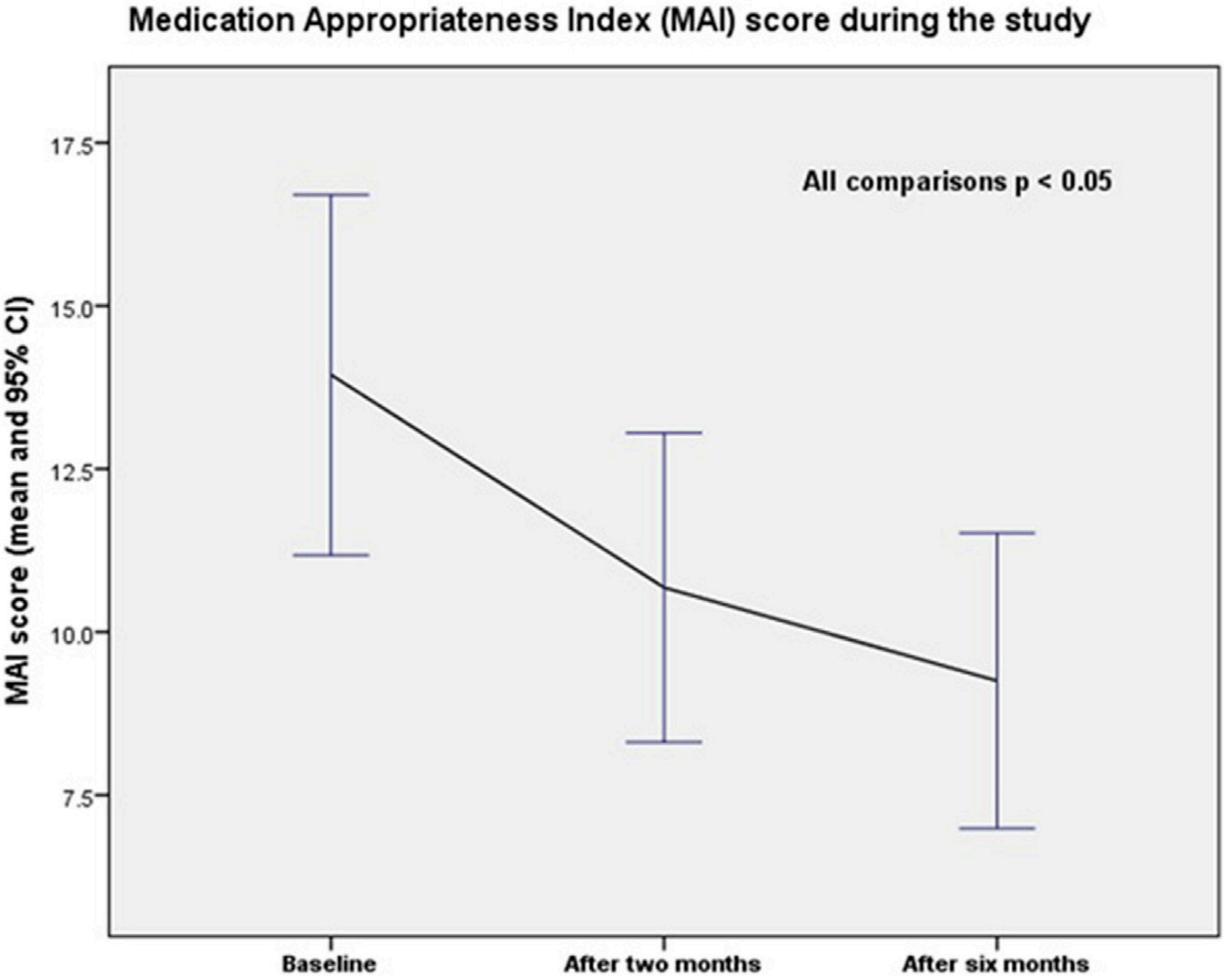
Medication Appropriateness Index (MAI) score during the study.

The average medication appropriateness index (MAI) score per patient decreased significantly during the study (p = 0.000 for all comparisons). The MAI score declined from 13.9 (SD = 15.2) at baseline to 10.7 (SD = 13.0) at 2 months and to 9.3 (SD = 12.5) at 6 months (end of the study). The differences were statistically significant (baseline vs. 2 months; p = 0.000; baseline vs. 6 months; p = 0.000; and 2 months vs. 6 months; p = 0.000). Changes in the mean MAI score per patient over the study period are shown in Figure 3.

The calculated effect size (Cohen’s d) was −0.361 between baseline and the end of the study, with a 95% confidence interval (CI) of −0.723 to −0.002. The number needed to treat (NNT) was 4.9.

The Anticholinergic Burden score also decreased significantly during the study (p = 0.001 for baseline vs. 6 months; p = 0.000 for baseline vs. 2 months; p = 0.240 for 2 months vs. 6 months), from 3.3 (SD = 4.3) at baseline to 2.8 (SD = 3.6) at 2 months, and remaining at 2.8 (SD = 3.8) at 6 months.

The number of GP visits per patient was non-significantly lower in the 3 months before the study (mean 4.24, SD = 3.5) than in the 3 months after the study began (mean 4.20, SD 4.1).

Clinical pharmacists reduced costs by EUR 456,619 (including discontinuation of 7×DDIs, 145 dose adjustments, nine duplicate discontinuations, 67 initiations for untreated conditions, 48 non-approved medication discontinuations, and 282 other interventions). This results in a return on investment *(ROI)* of EUR 22.3 for every EUR 1 invested. Even with additional sensitivity analysis, including interventions priced 50% lower than the original estimates, the return on investment (ROI) would be approximately 10:1.

### 3.3 Secondary outcomes

Clinical pharmacists prescribed 264 prescriptions to 119 patients during the study (from 265 included in the protocol), with a mean of 2.23 prescriptions per patient. GPs accepted 242 prescriptions issued by clinical pharmacists, resulting in an acceptance rate of 91.3%. The most frequently prescribed medications were rosuvastatin (28 prescriptions), followed by the rosuvastatin/ezetimibe combination (15 prescriptions) and pantoprazole (12 prescriptions). The most common reasons for prescribing were dyslipidemia (56 prescriptions), deprescribing (48 prescriptions), arterial hypertension (52 prescriptions), diabetes (29 prescriptions), depression (22 prescriptions), neuropathic pain (14 prescriptions), and dementia (10 prescriptions).

According to the DRP (Drug-Related Problem) classification, 192 prescriptions (72.8%) addressed and resolved the problems, while 40 prescriptions (15.1%) involved partially solved issues, and 23 prescriptions (8.7%) had unresolved problems. By the end of the study, 222 prescriptions (83.8%) were continued, and GPs changed 27 prescriptions (10.2%). Adherence to treatment guidelines improved significantly during the study (72 vs. 236 prescriptions; 29.8% vs. 90.9%; p = 0.000). The effect size, expressed as an odds ratio (OR), for adherence was 25.7 (95% CI: 15.6–42.4).

According to the clinical pharmacists’ final medication review, 204 prescriptions (77%) were positively evaluated in terms of reaching clinical outcomes, while 26 prescriptions (9.8%) were partially positive, and 15 cases (5.7%) were negative. Only 22 prescriptions were not accepted by GPs—in 10 cases, the patient did not want to take the new prescription; in six cases, there was no data; and in six cases, no reasons for the change were provided.

Clinical pharmacists prescribed new medications after the second visit in 69 cases, with only four prescriptions not maintained over 6 months, resulting in a 94.2% acceptance rate. All results relating to the secondary outcomes are summarised in Supplementary Material-Data sheet 1.

### 3.4 Tertiary outcomes

A total of 253 medications out of 264 were prescribed through the defined protocol. Only 11 prescriptions were issued outside the protocols (for nociceptive pain, osteopenia, and insomnia), as GPs specified these medications in the CPA. The majority of medications were prescribed (and also deprescribed) according to the deprescribing protocol (64 cases, 25.3%), followed by the dyslipidemia protocol (55 cases, 21.7%), arterial hypertension (32 cases, 12.6%), depression (25 cases, 9.9%), kidney and liver dose adjustments (21 cases, 8.3%), type II diabetes (23 cases, 9.1%), neuropathic pain (15 cases, 5.9%), dementia (9 cases, 3.6%), gout (6 cases, 2.4%), and asthma (3 cases, 1.2%).

Of the 253 medications prescribed through the protocols, GPs accepted 234, with only 18 prescriptions not accepted (acceptance rate: 92.5%). At the end of the study, 166 prescriptions accepted by GPs achieved the predefined outcomes for the conditions specified in the protocols (170 prescriptions, 73% of accepted prescriptions). According to the DRP classification, 169 prescriptions were resolved according to protocols (67%), followed by 49 prescriptions that were partially resolved (19%) and 27 prescriptions that remained unresolved (11%). Combining solved and partially resolved problems means that 86% of DRPs were addressed. The most common partially resolved problems were associated with improved outcomes when the target value was not achieved (e.g., a 5-point reduction on the PHQ-9, lower HbA1c levels). The number of prescriptions reaching the predefined clinical outcomes was significantly higher at the study’s end than at baseline (170 vs. 16; 70.8% vs. 6.4%; p = 0.000). The effect size (OR) for adherence to treatment guidelines was 33.9 (95% CI: 19.1–60.4). The results were also statistically significant for arterial hypertension, deprescribing, dyslipidaemia, depression, and kidney and liver dose adjustments (p = 0.000), and less significant for neuropathic pain (p = 0.005) and diabetes mellitus (p = 0.034).

All of the tertiary outcomes are summarised in Supplementary Material-Data sheet 2.

## 4 Discussion

This is the first nationally supported study to include pharmacists as prescribers outside of English-speaking countries, and the first in Europe outside the United Kingdom focused on primary care. In this context, the results are broadly applicable to other healthcare systems, developing interprofessional collaboration between GPs and clinical pharmacists focused on clinical pharmacist prescribing.

A similar pilot project was undertaken in Europe, although only in the United Kingdom, when pharmacist prescribers were developed and integrated into the United Kingdom healthcare system in 2003 (Tonna et al., 2007). They initiated collaboration with the CPA document, which enabled clinical pharmacists to prescribe. This collaboration became a standard of care in the United Kingdom. The authors reported positive outcomes during development, including patient views and adherence to treatment guidelines (Tonna et al., 2007). Another paper reported that all stakeholders, including GPs, supported pharmacists as dependent prescribers. However, they also noted that GPs expressed concerns about pharmacists’ independent prescribing. The authors suggested that pharmacists must develop new competencies and provide sufficient explanations to patients before the first consultation (Stewart et al., 2009). This development led to independent pharmacist prescribing, which was implemented in the United Kingdom in 2006, introducing additional competencies for independent prescribers (Tonna et al., 2007).

The first significant finding of our study is that pharmacist prescribers positively impact PROs, including quality of life and medication appropriateness. There is limited data on the impact of medication review on quality of life. One systematic review, including 31 randomised controlled trials, showed minimal effect on quality of life (Huiskes et al., 2017). The authors also noted the poor quality of the data and called for further studies on this topic (Huiskes et al., 2017). Previously, a small Slovenian prospective study, which included only 24 patients, also reported a positive impact of clinical pharmacists’ interventions in medication review on quality of life (Stuhec et al., 2019b). The researchers found that, after 2 months in a Slovenian nursing home, the total number of PIMs and DDIs was significantly decreased, and quality of life increased (p < 0.05). The clinical pharmacist did not have prescribing rights but provided medication review, which has been reimbursed nationally since 2017 (Stuhec, 2021; Stuhec et al., 2019b). Our study demonstrated that medication reviews, including pharmacist prescribers, lead to improved quality of life. We also observed a very positive impact on the MAI, with a moderate effect size, consistent with a systematic review published on this subject (Riordan et al., 2016).

The second important finding relates to the high prescription acceptance rate by GPs (91.3%), indicating successful collaboration. This can be explained by the long-term cooperation between clinical pharmacists and GPs in these primary care settings, where clinical pharmacists have worked for many years as ambulatory pharmacists providing medication reviews (Stuhec, 2021). This acceptance rate was considerably higher than that observed for the medication review service in Slovenia (90% vs. 50%). We propose the main reason for this is the close collaboration with GPs and the active communication maintained throughout the study. By contrast, medication reviews in Slovenia, usually do not include subsequent monitoring of patients as was the case in our study (Stuhec, 2021). This collaboration is essential to expand prescribing rights to non-medical professionals, such as clinical pharmacists. Clinical pharmacists assisted GPs in taking over some tasks, which has also been positively reported in primary care settings in the United Kingdom (Hasan Ibrahim et al., 2022). In this study, the United Kingdom involved 203 general practices; approximately two-thirds of GPs (62.4%, n = 126) reported that pharmacists were qualified as independent prescribers, and 83.6% believed that clinical pharmacists possessed sufficient skills to provide safe and effective treatment. Most GPs (>85%) expressed largely positive attitudes towards collaboration with practice-based pharmacists and noted that this collaboration could enhance cooperation between GPs and pharmacists (Hasan Ibrahim et al., 2022).

In our study, we found that clinical pharmacists prescribed the most medications for dyslipidaemia, arterial hypertension, diabetes, and depression. Deprescribing also represented an important aspect of care. These results align with the US collaborative care model, which utilises the CPA document, where clinical pharmacists prescribe medications most frequently for these conditions (Choe et al., 2018; American Pharmacists Ass ociation (APhA); Finley et al., 2003). We also demonstrated that adherence to treatment guidelines improved significantly, which is consistent with our previous studies, including medication review in Slovenia (Stuhec, 2021).

The third important finding relates to clinical outcomes, which improved significantly during our prospective study. Clinical pharmacist prescribers achieved positive clinical outcomes in nearly 70% of patients, indicating noteworthy results. Our results are in line with previous studies on this type of collaboration (Weeks et al., 2016; Finley et al., 2003). Additionally, the findings showed a very high acceptance rate when pharmacists utilised protocols (92%), further indicating that protocols benefit pharmacists and GPs. This suggests that clinical pharmacist prescribers, in collaboration with GPs, could constitute an essential team for enhancing clinical outcomes. Improvements in clinical outcomes were observed across almost all protocols, particularly in diabetes, dyslipidaemia, depression, and arterial hypertension. In addition, collaboration with a clinical pharmacist resolved or partially resolved 86% of DRPs, indicating that, even when target outcomes were not fully achieved, this collaboration still led to significant improvements in many patients (e.g., patients with depression who showed a response but did not achieve remission). This suggests that a longer study may demonstrate an even higher proportion of patients reaching target outcomes.

In our study, clinical pharmacists monitored patients with complex comorbidities, often involving multiple medications. These findings are especially significant in this context and demonstrate that clinical pharmacists and prescribers can effectively manage various conditions and complex cases. Many patients with these comorbidities do not achieve the recommended target values (Kessler et al., 2003; Lech et al., 2022; Smolders et al., 2009; Redon et al., 2016; Shrivastav et al., 2018). Our study showed that clinical pharmacist prescribers improve the percentage of patients who reach target values across different conditions. This could be valuable for managing chronic conditions in Slovenia and beyond. For example, in depression treatment, clinical pharmacists helped 19/25 (76%) reach target values, compared with only 5% at baseline. This aligns with other studies showing that clinical pharmacists can effectively manage depression in primary care and improve its current management (Finley et al., 2003). Similar improvements were seen in our study for arterial hypertension, diabetes, and dyslipidemia, where clinical pharmacist prescribers increased the percentage of patients achieving target values to 70%, 40%, and 48%, respectively. This approach could be evaluated in future randomised prospective studies.

This study also shows that monitoring by clinical pharmacists is beneficial, as they are able to follow patients and assess long-term outcomes. In Slovenia, medication reviews are based on a single assessment rather than ongoing monitoring, which was the approach taken in our study (Stuhec, 2021). Our findings are consistent with the Committee of Ministers’ Resolution CM/Res (2020)3 on the Implementation of pharmaceutical care for the benefit of patients and health services, which supports monitoring by clinical pharmacists (Committee of Ministers Resolution CM/Res, 2020). These results are therefore valuable for the implementation of medication reviews, which have been successfully introduced in Slovenia but could be further developed by shifting from single reviews to ongoing monitoring. Such a change would strengthen collaboration between GPs and clinical pharmacists. As we have shown, this could improve clinical outcomes. This would mean that subsequent appointments, after the initial one, would be initiated by the clinical pharmacist rather than the GP. This study also demonstrated positive pharmacoeconomic outcomes. Based on an additional sensitivity analysis—assuming intervention costs were 50% lower than the original estimates—the return on investment (ROI) would be approximately 10:1, thereby strengthening the case for reimbursement by the Health Insurance Institute of Slovenia. Financial coverage is crucial, and the Health Insurance Institute of Slovenia should therefore consider reimbursing this service (including additional reimbursement for prescribing).

In addition, the study revealed some other significant findings, such as the impact on the number of medications, PIMs, and DDIs. The number of medications did not change significantly, which could be attributed to GP referrals. GPs prescribed medications for known conditions but referred patients for whom they did not initiate new medications; therefore, pharmacists prescribed and monitored these patients. Conversely, the number of PIMs and type D DDIs decreased significantly, representing a positive outcome. In our study, clinical pharmacists also successfully deprescribed many medications, particularly proton pump inhibitors and benzodiazepines. Proton pump inhibitors are among the most prescribed medication groups worldwide and are often overprescribed. In this context, clinical pharmacist prescribers play an essential role in rational deprescribing within this population (Muheim et al., 2021). The number of patients visiting the general practice did not significantly differ. However, this was limited to a 3-month monitoring period.

These results align with a study on pharmacist prescribers in the United Kingdom (Alharthi et al., 2023). In this study, researchers examined medications prescribed by clinical pharmacist prescribers in 284 of 370 residents across United Kingdom care homes. They analysed the relationship between the number of medicines stopped and various contextual factors (such as the number of residents cared for, pharmacist employment within the associated medical practice, previous care home experience, hours active within the trial, years of experience as a pharmacist, and prescriber status). The authors found that the number of residents and employment of pharmacist independent prescribers within a medical practice were positive predictors of deprescribing (Alharthi et al., 2023). The positive impact of clinical pharmacists on reducing PIMs and DDIs was previously demonstrated in medication reviews conducted in Slovenia (Stuhec, 2021).

This study has several limitations that should be acknowledged. Firstly, it was not a randomised controlled trial (comparison with usual care), which limits the generalisability of the findings. This design was chosen because the study population is multimorbid, with many medication-related issues, and is comparable in clinical characteristics to populations in real settings. Additionally, comparable pilot studies have also employed this approach (Cardwell et al., 2020). Moreover, we aimed to reflect real clinical situations with minimal exclusion criteria. Quality of life is a recommended outcome measure in elderly patients with multiple comorbidities, as it reflects daily clinical practice. This could also be considered one of the strengths of our study, as it included quality-of-life measurements, which are recommended in studies involving elderly patients with multimorbidity (Stuhec et al., 2019b). Another limitation relates to the relatively small effect size, which was influenced by the limited number of primary care settings and clinical pharmacists involved. However, sample size calculations mitigated this, ensuring sufficient statistical power for the study. We should also mention possible selection bias, as GPs referred patients to a clinical pharmacist prescriber. This was inherent to the nature of the pilot trial. Since pharmacist prescribers have not yet been studied at the national level, our starting point was the existing medication review service in Slovenia, which is already established. Additionally, selection bias may be associated with the four primary care settings chosen, which were selected based on previous good collaboration between clinical pharmacists and GPs in these institutions—an essential factor for conducting this pilot project. Another limitation relates to the prescription type, as GPs must confirm each prescription. This was one of the most significant limitations at the outset of the project, as clinical pharmacists do not have prescribing rights in Slovenia, and this should have been planned accordingly. The Slovenian National Medical Ethics Committee approved the ethical aspect of prescribing by clinical pharmacists, which was essential for this study. The study did not assess the level of trust between GPs and clinical pharmacists. We are currently conducting qualitative research, including semi-structured interviews with 16 participants (patients, GPs, and pharmacists). The findings will be published in a subsequent paper.

On the other hand, our pilot trial represents the first national pilot project on pharmacist prescribers in Europe outside the United Kingdom, providing essential information for all GPs and clinical pharmacists who aim to develop this type of collaboration across Europe and beyond. This pilot could serve as a basis for systemic reimbursement and legislative changes in Slovenia, enabling clinical pharmacists’ prescribing rights, similar to those in the US. Additionally, we have developed a CPA document and protocols that could be implemented nationally and adapted for use in other countries. A further essential step is for the Slovene Chamber of Pharmacy to establish the necessary education and certification programmes for pharmacist prescribers in Slovenia. Similar competencies have already been developed for clinical pharrmacists in Slovenia (Stuhec, 2021).

In conclusion, this is the first national study describing the impact of pharmacist prescribers in primary care settings outside the United Kingdom. The study demonstrates that prescriptions made by clinical pharmacists in collaboration with GPs, as specified in the CPA, improved patient-reported outcomes (PROs) and clinical outcomes for predefined chronic non-communicable conditions and showed positive economic results. The results of this pilot are broadly applicable in different settings and could promote the development of collaborative practice models in many countries. Further qualitative research involving patients, GPs, and clinical pharmacists is necessary to gather additional insights, such as acceptability and enabling actions for systematic implementation in national healthcare systems.

## Data Availability

The datasets presented in this study can be found in online repositories. The names of the repository/repositories and accession number(s) can be found in the article/Supplementary Material.

## References

[B1] Alharthi M. Scott S. Alldred D. P. Holland R. Hughes C. Birt L. (2023). Pharmacist-independent prescriber deprescribing in UK care homes: contextual factors associated with increased activity. Br. J. Clin. Pharmacol. 89 (4), 1509–1513. 10.1111/bcp.15643 36516106

[B2] Alliabi F. J. A. Jaber A. A. S. Jallo M. K. I. Baig M. R. (2022). Adherence of physicians to evidence-based management guidelines for treating type 2 diabetes and atherosclerotic cardiovascular disease in Ajman, United Arab Emirates. BMC Prim. Care 23 (1), 70. 10.1186/s12875-022-01672-4 35392814 PMC8988318

[B3] Alsaeed B. A. Hall J. Keers R. N. (2025). Exploring non-medical prescribing for patients with mental illness: a scoping review. BMC Psychiatry 25 (1), 504. 10.1186/s12888-025-06938-6 40389900 PMC12090459

[B4] American Pharmacists Association (APhA) Collaborative practice agreements and pharmacists patient care services. Available online at: https://www.aphafoundation.org/collaborative-practice-agreements (Accessed August 18, 2025).

[B5] American Pharmacists Association (APhA) Collaborative practice agreements and pharmacists patient care services. Available online at: https://www.aphafoundation.org/collaborative-practice-agreements (Accessed August 29, 2025).

[B6] Anticholinergic burden calculator. Available online at: https://www.acbcalc.com/ (Accessed August 25, 2025).

[B7] Bennie M. Santa-Ana-Tellez Y. Galistiani G. F. Trehony J. Despres J. Jouaville L. S. (2024). The prevalence of polypharmacy in older Europeans: a multi-national database study of general practitioner prescribing. Br. J. Clin. Pharmacol. 90 (9), 2124–2136. 10.1111/bcp.16113 38812250

[B8] Cardwell K. Smith S. M. Clyne B. McCullagh L. Wallace E. Kirke C. (2020). Evaluation of the general practice pharmacist (GPP) intervention to optimise prescribing in Irish primary care: a non-randomised pilot study. BMJ Open 10 (6), e035087. 10.1136/bmjopen-2019-035087 32595137 PMC7322285

[B9] Carter M. Chapman S. Rogers P. Watson M. (2024). Practice pharmacists and their influence on prescribing in UK general practice: a cross-sectional study. Int. J. Pharm. Pract. 32 (1), 69–75. 10.1093/ijpp/riad075 38006341

[B10] Chisholm-Burns M. A. Kim Lee J. Spivey C. A. Slack M. Herrier R. N. Hall-Lipsy E. (2010). US pharmacists' effect as team members on patient care: systematic review and meta-analyses. Med. Care 48, 923–933. 10.1097/MLR.0b013e3181e57962 20720510

[B11] Choe H. M. Lin A. T. Kobernik K. Rockey N. G. Ashjian E. J. Dorsch M. P. (2018). Michigan pharmacists transforming care and quality: developing a statewide collaborative of physician organizations and pharmacists to improve quality of care and reduce costs. J. Manag. Care Spec. Pharm. 24 (4), 373–378. 10.18553/jmcp.2018.24.4.373 29578853 PMC10397673

[B12] Committee of Ministers Resolution CM/Res (2020). 3 on the implementation of pharmaceutical care for the benefit of patients and health services. Available online at: https://go.edqm.eu/ResPhCare20203 (Accessed August 29, 2025).

[B13] Cope L. C. Abuzour A. S. Tully M. P. (2016). Nonmedical prescribing: where are we now? Ther. Adv. Drug Saf. 7 (4), 165–172. 10.1177/2042098616646726 27493720 PMC4959632

[B14] European health information gateway (2025). Copenhagen: World Health Organization. Available online at: https://gateway.euro.who.int/en/indicators/hlthres_71-general-practitioners-per-10-000/#id=27994 (Accessed August 29, 2025).

[B15] Finley P. R. Rens H. R. Pont J. T. Gess S. L. Louie C. Bull S. A. (2003). Impact of a collaborative care model on depression in a primary care setting: a randomized controlled trial. Pharmacotherapy 23 (9), 1175–1185. 10.1592/phco.23.10.1175.32760 14524649

[B16] Gorup E. C. Šter M. P. (2017). Number of medications or number of diseases: what influences underprescribing? Eur. J. Clin. Pharmacol. 73 (12), 1673–1679. 10.1007/s00228-017-2336-x 28920183

[B17] Hanlon J. Schmader K. Samsa G. Weinberger M. Uttech K. M. Lewis I. K. (1992). A method for assessing drug therapy appropriateness. J. Clin. Epidemiol. 45, 1045–1051. 10.1016/0895-4356(92)90144-c 1474400

[B18] Hasan Ibrahim A. S. Barry H. E. Hughes C. M. (2022). General practitioners' experiences with, views of, and attitudes towards, general practice-based pharmacists: a cross-sectional survey. BMC Prim. Care 23 (1), 6. 10.1186/s12875-021-01607-5 35172734 PMC8759266

[B19] Horvat N. Kos M. (2016). Development and validation of the Slovenian drug-related problem classification system based on the PCNE classification V 6.2. Int. J. Clin. Pharm. 38 (4), 950–959. 10.1007/s11096-016-0320-7 27255777

[B20] Huiskes V. J. Burger D. M. van den Ende C. H. van den Bemt B. J. (2017). Effectiveness of medication review: a systematic review and meta-analysis of randomized controlled trials. BMC Fam. Pract. 18 (1), 5. 10.1186/s12875-016-0577-x 28095780 PMC5240219

[B21] Kessler R. C. Berglund P. Demler O. Jin R. Koretz D. Merikangas K. R. (2003). The epidemiology of major depressive disorder: results from the national Comorbidity Survey Replication (NCS-R). JAMA 289 (23), 3095–3105. 10.1001/jama.289.23.3095 12813115

[B22] Komwong D. Greenfeld G. Zaman H. Majeed A. Hayhoe B. (2018). Clinical pharmacists in primary care: a safe solution to the workforce crisis? J. R. Soc. Med. 111, 120–124. 10.1177/0141076818756618 29480743 PMC5900835

[B23] Lech S. Herrmann W. Trautmann S. Schwantes U. Gellert P. Behr J. (2022). Depression in primary care and the role of evidence-based guidelines: cross-sectional data from primary care physicians in Germany. BMC Health Serv. Res. 22 (1), 1279. 10.1186/s12913-022-08631-w 36280876 PMC9594952

[B24] Lee A. J. Boro M. S. Knapp K. K. Meier J. L. Korman N. E. (2002). Clinical and economic outcomes of pharmacist recommendations in a Veterans Affairs medical center. Am. J. Health Syst. Pharm. 59 (21), 2070–2077. 10.1093/ajhp/59.21.2070 12434719

[B25] Maher R. L. Hanlon J. Hajjar E. R. (2014). Clinical consequences of polypharmacy in elderly. Expert Opin. Drug Saf. 13, 57–65. 10.1517/14740338.2013.827660 24073682 PMC3864987

[B26] Mann N. K. Mathes T. Sönnichsen A. Pieper D. Klager E. Moussa M. (2023). Potentially inadequate Medications in the Elderly: PRISCUS 2.0. Dtsch. Arztebl Int. 120 (1-2), 3–10. 10.3238/arztebl.m2022.0377 36507719 PMC10035347

[B27] McAlister F. A. Majumdar S. R. Padwal R. S. Fradette M. Thompson A. Buck B. (2014). Case management for blood pressure and lipid level control after minor stroke: PREVENTION randomized controlled trial. CMAJ 186 (8), 577–584. 10.1503/cmaj.140053 24733770 PMC4016053

[B28] Midão L. Giardini A. Menditto E. Kardas P. Costa E. (2018). Polypharmacy prevalence among older adults based on the survey of health, ageing and retirement in Europe. Arch. Gerontol. Geriatr. 78, 213–220. 10.1016/j.archger.2018.06.018 30015057

[B29] Muheim L. Signorell A. Markun S. Chmiel C. Neuner-Jehle S. Blozik E. (2021). Potentially inappropriate proton-pump inhibitor prescription in the general population: a claims-based retrospective time trend analysis. Ther. Adv. Gastroenterol. 14, 1756284821998928. 10.1177/1756284821998928 33948109 PMC8053831

[B30] Nakhla N. Leung V. Schwartz K. L. (2024). Expansion of pharmacist prescribing could help improve health care access and quality. Can. Fam. Physician 70 (7-8), 441–443. 10.46747/cfp.700708441 39122432 PMC11328721

[B31] PCNE PCNE statement on medication review 2013. Available online at: https://www.pcne.org/upload/files/150_20160504_PCNE_MedRevtypes.pdf (Accessed August 25, 2025).

[B32] Pharmacist prescriber scope of practice in New Zealand. Available online at: https://pharmacycouncil.org.nz/wp-content/uploads/2021/04/Pharmacist-Prescriber-Scope-of-Practice-reviewed-Oct-17.pdf (Accessed August 25, 2025).

[B33] Raghunandan R. Marra C. A. Tordoff J. Smith A. (2021). Examining non-medical prescribing trends in New Zealand: 2016-2020. BMC Health Serv. Res. 21 (1), 418. 10.1186/s12913-021-06435-y 33941188 PMC8094524

[B34] Redon J. Mourad J. J. Schmieder R. E. Volpe M. Weiss T. W. (2016). Why in 2016 are patients with hypertension not 100% controlled? A call to action. J. Hypertens. 34 (8), 1480–1488. 10.1097/HJH.0000000000000988 27270186

[B35] Riordan D. O. Walsh K. A. Galvin R. Sinnott C. Kearney P. M. Byrne S. (2016). The effect of pharmacist-led interventions in optimising prescribing in older adults in primary care: a systematic review. SAGE Open Med. 4, 2050312116652568. 10.1177/2050312116652568 27354917 PMC4910534

[B36] Shrivastav M. Gibson W. Jr Shrivastav R. Elzea K. Khambatta C. Sonawane R. (2018). Type 2 diabetes management in primary care: the role of retrospective, professional continuous glucose monitoring. Diabetes Spectr. 31 (3), 279–287. 10.2337/ds17-0024 30140145 PMC6092883

[B37] Smolders M. Laurant M. Verhaak P. Prins M. van Marwijk H. Penninx B. (2009). Adherence to evidence-based guidelines for depression and anxiety disorders is associated with recording of the diagnosis. Gen. Hosp. Psychiatry 31 (5), 460–469. 10.1016/j.genhosppsych.2009.05.011 19703640

[B38] Stewart D. C. George J. Bond C. M. Diack H. L. McCaig D. J. Cunningham S. (2009). Views of pharmacist prescribers, doctors and patients on pharmacist prescribing implementation. Int. J. Pharm. Pract. 17 (2), 89–94. 10.1211/ijpp/17.02.0003 20214256

[B39] Stuhec M. (2021). Clinical pharmacist consultant in primary care settings in Slovenia focused on elderly patients on polypharmacy: successful national program from development to reimbursement. Int. J. Clin. Pharm. 43 (6), 1722–1727. 10.1007/s11096-021-01306-2 34228266

[B40] Stuhec M. Gorenc K. Zelko E. (2019a). Evaluation of a collaborative care approach between general practitioners and clinical pharmacists in primary care community settings in elderly patients on polypharmacy in Slovenia: a cohort retrospective study reveals positive evidence for implementation. BMC Health Serv. Res. 19 (1), 118. 10.1186/s12913-019-3942-3 30760276 PMC6375190

[B41] Stuhec M. Bratović N. Mrhar A. (2019b). Impact of clinical pharmacist's interventions on pharmacotherapy management in elderly patients on polypharmacy with mental health problems including quality of life: a prospective non-randomized study. Sci. Rep. 9 (1), 16856. 10.1038/s41598-019-53057-w 31728029 PMC6856189

[B42] Tonna A. P. Stewart D. West B. McCaig D. (2007). Pharmacist prescribing in the UK - a literature review of current practice and research. J. Clin. Pharm. Ther. 32 (6), 545–556. 10.1111/j.1365-2710.2007.00867.x 18021331

[B43] Urbańczyk K. Guntschnig S. Antoniadis V. Falamic S. Kovacevic T. Kurczewska-Michalak M. (2023). Recommendations for wider adoption of clinical pharmacy in Central and Eastern Europe in order to optimise pharmacotherapy and improve patient outcomes. Front. Pharmacol. 14, 1244151. 10.3389/fphar.2023.1244151 37601045 PMC10433912

[B44] von Elm E. Altman D. G. Egger M. Pocock S. J. Gøtzsche P. C. Vandenbroucke J. P. (2008). The strengthening the reporting of Observational Studies in Epidemiology (STROBE) statement: guidelines for reporting observational studies. J. Clin. Epidemiol. 61 (4), 344–349. 10.1016/j.jclinepi.2007.11.008 18313558

[B45] Weeks G. George J. Maclure K. Stewart D. (2016). Non-medical prescribing *versus* medical prescribing for acute and chronic disease management in primary and secondary care. Cochrane Database Syst. Rev. 11 (11), CD011227. 10.1002/14651858.CD011227.pub2 27873322 PMC6464275

