# Supplementary material for: Clinical pharmacist prescriber in primary care in Slovenia: prospective non-randomised interventional study focused on clinical outcomes and quality of life
Source: Front Pharmacol. 2025 Sep 15;16:1690480. doi: 10.3389/fphar.2025.1690480 (PMC12477041; doi:10.3389/fphar.2025.1690480)
Supplement: Supplementary file 1 [file DataSheet4.docx]

**Arterial Hypertension – Protocol**

**Treatment goals:**

| Achieving an individually determined target blood pressure range (Outcome N#1) |
| --- |
| Improving quality of life (Outcome N#2) |

**Process:**

Monitoring of clinical outcomes

Measurements:

1. BP > 140/90 mmHg (18–79 years) or 160/90 mmHg (≥80 years)

2. Blood pressure within the target range after I–II months

3. Blood pressure within the target range after VI months

Patient enters the prospective study upon physician’s referral (signs consent)

End of study

(6 months)

**Outcomes of monitoring and measurement methods**

| **Clinical monitoring outcome** | **Target BP values** | **Measurement time (t)** | **Variable** |
| --- | --- | --- | --- |
| Controlled blood pressure (Outcome N#1) |  | 0, 1, 2, 6 months | Δ difference, % of patients with uncontrolled BP |
| **Humanistic monitoring outcome** |  |  |  |
| Quality of life (Outcome N#2) | EQ-5D | 0, 2, 6 months | Number (absolute), Δ |
| Additional: Treatment in accordance with the 2023 Guidelines for the management of arterial hypertension | State before | State after | Δ difference in the number of patients treated in accordance with the guidelines and % |

**Tasks of the pharmacist**

The clinical pharmacist may prescribe a new medicine (antihypertensive) and adjust doses (in accordance with the Protocol) as well as additional medicines, if necessary and permitted by the Protocol. The pharmacist may also consult the patient by phone. Included are patients with an established diagnosis of arterial hypertension, newly diagnosed patients, and those whose prescribed therapy has not achieved target blood pressure values considering age and comorbidities (Table 1).

Tabela 1: Target blood pressure values (European Guidelines fo hypertention management 2023)

| Age (y) | Target SBP (mmHg) | | | | | Target DBP  (mmHg) |
| --- | --- | --- | --- | --- | --- | --- |
|  | Arterial blood pressure | + Diabetes melitus | + Chronic kidney disease | + Coronary disease | + Stroke/Transient ischaemic attack |  |
| 18-65 | 120-130 | 120-130 | < 130 | 120-130 | 120-130 | 70-79 |
| 65-79 | < 140 | < 140 | < 140 | < 140 | < 140 | 70-79 |
| 80 or more | 140-159 | 140-159 | 140-159 | 140-159 | 140-159 | 70-79 |
| Target DBP  (mmHg) | 70-79 | 70-79 | 70-79 | 70-79 | 70-79 |  |

*SBP – systolic blood pressure; DBP – diastolic blood pressure; AH – arterial hypertension; DM – diabetes mellitus; CKD – chronic kidney disease; CVI – ischemic stroke; TIA – transient ischemic attack; 1 – if well tolerated 130/80 mmHg; 2 – if well tolerated 130-139/80 mmHg.*

In the consultant pharmacist’s office, the clinical pharmacist measures blood pressure on both upper arms (3 measurements at 1-minute intervals, average of the last 2). In elderly patients or those with comorbidities/complications, standing measurements are also performed to rule out orthostatic hypotension.

After pharmacotherapy optimization, the patient performs home BP measurements for 7 (or minimum 3) consecutive days in the morning and evening (average of two measurements at 1-minute intervals). During follow-up visits in the consultant pharmacist’s office, the clinical pharmacist reviews home BP measurements and measures BP in the office.

If the pharmacist and/or the patient assess that the condition has significantly worsened, the pharmacist refers the patient to a physician.

**Guidelines**

Guidelines: 2023 ESH Guidelines for the management of arterial hypertension. Available at:

<https://journals.lww.com/jhypertension/fulltext/2024/01000/2023_esh_guidelines_for_the_management_of_arterial.29.aspx>

**Depression – Protocol**

**Treatment Goals:**

| Achieving remission (Outcome N#1) |
| --- |
| Achieving response to treatment (Outcome N#2) |
| Improving quality of life (Outcome N#3) |

**Process:**

**Outcomes of monitoring and measurement methods**

| **Clinical Outcome Monitoring** | **Questionnaire** | **Measurement Time (t)** | **Variable** |
| --- | --- | --- | --- |
| Achieving Remission (Outcome N#1) | PHQ-9 score less than 5 | 0, 1, 2, 6 months | Number of points (absolute), Δ difference, % of patients in remission |
| Achieving Response (Outcome N#2) | PHQ-9 score less than 10 | After 1 month | Number of points (absolute), Δ difference, % of patients responding |
| **Humanistic Outcome Monitoring** |  |  |  |
| Quality of Life (Outcome N#3) | EQ-5D | 0, 2, 6 months | Number of points (absolute), Δ A difference in the number treated in accordance with guidelines and % |
| Additional: Treatment in accordance with NICE 2022 guidelines | **/** | Pre-treatment | Post-treatment |

**Tasks of the pharmacist**

The clinical pharmacist can prescribe a new drug (antidepressant) and modify dosages (in accordance with the Protocol) and additional drugs, if this is necessary and the Protocol allows it. The pharmacist can also consult the patient by telephone. Included are patients who have an established diagnosis of depression and who are not in remission and/or do not respond to treatment. If the pharmacist and/or patient determines that the condition has significantly worsened, the pharmacist refers the patient to a physician.

**Guidelines**

Guidelines (NICE 2022): Available at <https://www.nice.org.uk/guidance/ng222>

**Dyslipidemia - Protocol**

**Treatment Goals:**

| Achieving individually determined target LDL values (Outcome N#1) |
| --- |
| Improving quality of life (Outcome N#2) |

**Proces:**

**Outcomes of monitoring and measurement methods**

| **Clinical Outcome Monitoring** | **Questionnaire** | **Measurement Time (t)** | **Variable** |
| --- | --- | --- | --- |
| Achieving target LDL values (Outcome N#1) | **/** | 0, 2, 6 months | Δ difference, % of patients who achieved the target LDL cholesterol value |
| **Humanistic Outcome Monitoring** |  |  |  |
| Quality of Life (Outcome N#2) | EQ-5D | 0, 2, 6 months | Number of points (absolute), Δ Δ difference in the number treated in accordance with guidelines and % |
| Additional: Treatment in accordance with guidelines | **/** | Pre-treatment | Post-treatment |

**Tasks of the pharmacist**

The clinical pharmacist can prescribe a new drug (statin, ezetimibe) and/or modify dosages (in accordance with the Protocol). The pharmacist can also consult the patient by telephone. Included are patients who have an established diagnosis of dyslipidemia and who have not reached the target LDL cholesterol value based on the estimated cardiovascular risk (CVR) . The target LDL value in certain patients in secondary prevention may be lower than <1.4 mmol/L and is individually determined based on risk factors.

**Guidelines**

ESC Guidelines for the Prevention of Cardiovascular Diseases 2021; Available at: <https://www.escardio.org/Guidelines/Clinical-Practice-Guidelines/CVD-Prevention-Guidelines>

**Deprescribing – Protocol**

**Treatment Goals:**

| 1. Medication is prescribed in accordance with a clear indication - (Outcome N#1) |
| --- |
| 1. Medications are prescribed in accordance with Priscus recommendations - (Outcome N#2) |
| 1. Improvement in quality of life (Outcome N#3) |

**Process:**

**Outcomes of monitoring and measurement methods**

| **Clinical Outcome Monitoring** | **Methods** | **Measurement Time (t)** | **Variable** |
| --- | --- | --- | --- |
| Medication is prescribed in accordance with a clear indication - (Outcome N#1) | SmPC, documentation | 0, 2, 6 months | Absolute difference, Δ difference, % of patients prescribed in accordance with recommendations |
| Medications are prescribed in accordance with Priscus recommendations - (Outcome N#2) | Priscus 2.0 list (Table 2) | 0, 2, 6 months | Absolute difference, Δ difference, % of patients prescribed in accordance with recommendations |
| **Humanistic Outcome Monitoring** |  |  |  |
| Quality of Life (Outcome N#3) | EQ-5D | 0, 2, 6 months | Number of points (absolute), Δ |

***SmPC (Summary of Product Characteristics). Definition: Deprescribing means stopping or reducing the dose.**

**Tasks of the pharmacist**

The clinical pharmacist can prescribe a new medication, modify dosages (in accordance with the Protocol), and prescribe additional medications if necessary and the Protocol allows it. The pharmacist can also consult the patient by telephone. Patients who have an established diagnosis and for whom deprescribing needs to be carried out are included.

If the pharmacist and/or patient determines that the condition has significantly worsened, the pharmacist refers the patient to a physician.

**Guidelines**

- Summaries of Product Characteristics (SmPC).
- Priscus 2.0 list. Mann NK, Mathes T, Sönnichsen A, Pieper D, Klager E, Moussa M, Thürmann PA. Potentially Inadequate Medications in the Elderly: PRISCUS 2.0. Dtsch Arztebl Int. 2023 Jan 9;120(1-2):3-10. <https://pmc.ncbi.nlm.nih.gov/articles/PMC10035347/>
