# Supplementary material for: Clinical pharmacist prescriber in primary care in Slovenia: prospective non-randomised interventional study focused on clinical outcomes and quality of life
Source: Front Pharmacol. 2025 Sep 15;16:1690480. doi: 10.3389/fphar.2025.1690480 (PMC12477041; doi:10.3389/fphar.2025.1690480)
Supplement: Supplementary file 2 [file DataSheet3.docx]

| Patient’s full name and personal identification number: | The form is attached to the referral for the consultant pharmacist. Communication between the physician and pharmacist is reciprocal. |
| --- | --- |
| Physician (full name and signature):  Date: | Consultant pharmacist (full name and signature):  Date: |
| The consultant pharmacist may, within the framework of the agreement, prescribe/discontinue medication and monitor pharmacotherapy (the authorization applies to all). The physician indicates what the consultant pharmacist is authorized to do (please mark below): | The consultant pharmacist prepares the ePrescription, which the physician reviews (approves, modifies, or cancels). The pharmacist records each consultation in the medical record, available to the physician. The pharmacist may also order laboratory tests related to the planned or prescribed therapy. |
| □ All pharmacotherapeutic groups of medicines from the list (Annex – mandatory to mark) | The consultant pharmacist may prescribe all medicines included in the list (all items in the Annex below) and optimize treatment for the specified indications. In this case, the pharmacist will review all groups and prescribe medication. The physician may mark one, several, or all groups. |
| □ Individual medications within these groups (Annex – mandatory to mark chosen groups) | Enter here only if you authorize the pharmacist solely for a specific medication within one of the groups, not the entire group (otherwise mark the entire medicine group from the Annex). |
| □ Specific medicationsa not included in the Annex but within the expertise of the consultant pharmacist | Please specify the medicication, indication, and monitoring method in detail (for groups or medicines not included in the Annex). |
| Specify particular details you wish to transfer to the consultant pharmacist: | E.g., allergies, when to necessarily call the physician, drug non-responsiveness, and other specifics. |
|  |  |

Annex: List of pharmacotherapeutic groups or conditions that the consultant pharmacist may prescribe (MUST be marked with an X):

|  | □ | **ALL GROUPS listed (numbers 2–11) – I authorize the pharmacist for prescribing, monitoring, and optimization of all groups** |
| --- | --- | --- |
|  | □ | Lipids not within target range (initiation and titration of oral medications) – diagnosis: dyslipidemia |
|  | □ | Neuropathic pain – adjustment of therapy (initiation and titration) – diagnosis: neuropathic pain |
|  | □ | Blood pressure not within target range (initiation and titration) – diagnosis: arterial hypertension |
|  | □ | Diabetes – HbA1c and/or blood glucose not within target range (initiation and titration) – diagnosis: type II diabetes |
|  | □ | Depression remission not achieved (initiation and titration) – diagnosis: depression |
|  | □ | Medications for dementia drugs (initiation and titration) – diagnosis: dementia |
|  | □ | Drugs for gout treatment (initiation and titration) – diagnosis: gout |
|  | □ | Dose adjustment according to renal and hepatic function – diagnosis: chronic kidney and/or chronic liver disease |
|  | □ | Deprescribing (within the framework of therapy optimization) |
|  | □ | Titration of medications for asthma treatment – diagnosis: asthma |

- The pharmacist may prescribe those medicines within individual groups as a general practitioner may
